# A CRISPR/Cas9-based vector system enables the fast breeding of selection-marker-free canola with *Rcr1*-rendered clubroot resistance

**DOI:** 10.1093/jxb/erad471

**Published:** 2023-11-22

**Authors:** Hao Hu, Yan Zhang, Fengqun Yu

**Affiliations:** Agriculture and Agri-Food Canada, Saskatoon Research and Development Centre, 107 Science Place, Saskatoon, SK, S7N 0X2, Canada; Agriculture and Agri-Food Canada, Saskatoon Research and Development Centre, 107 Science Place, Saskatoon, SK, S7N 0X2, Canada; Agriculture and Agri-Food Canada, Saskatoon Research and Development Centre, 107 Science Place, Saskatoon, SK, S7N 0X2, Canada; University of Trento, Italy

**Keywords:** *Brassica*, canola, cisgenesis, clubroot disease resistance, CRISPR/Cas9 vector, genome editing, intragenesis, introgression, *Plasmodiophora brassicae*, plant breeding, *Rcr1*

## Abstract

Breeding for disease resistance in major crops is of crucial importance for global food security and sustainability. However, common biotechnologies such as traditional transgenesis or genome editing do not provide an ideal solution, whereas transgenic crops free of selection markers such as cisgenic/intragenic crops might be suitable. In this study, after cloning and functional verification of the *Rcr1* gene for resistance to clubroot (*Plasmodiophora brassicae*), we confirmed that the genes *Rcr1*, *Rcr2*, *Rcr4*, and *CRa* from *Brassica rapa* crops and the resistance gene from *B. napus* oilseed rape cv. ‘Mendel’ on chromosome A03 were identical in their coding regions. We also determined that *Rcr1* has a wide distribution in *Brassica* breeding materials and renders potent resistance against multiple representative clubroot strains in Canada. We then modified a CRISPR/Cas9-based cisgenic vector system and found that it enabled the fast breeding of selection-marker-free transgenic crops with add-on traits, with selection-marker-free canola (*B. napus*) germplasms with *Rcr1*-rendered stable resistance to clubroot disease being successfully developed within 2 years. In the *B. napus* background, the intragenic vector system was able to remove unwanted residue sequences from the final product with high editing efficiency, and off-target mutations were not detected. Our study demonstrates the potential of applying this breeding strategy to other crops that can be transformed by *Agrobacterium*. Following the streamlined working procedure, intragenic germplasms can be developed within two generations, which could significantly reduce the breeding time and labor compared to traditional introgression whilst still achieving comparable or even better breeding results.

## Teaser

A modified CRISPR/Cas9-based vector system enables the fast breeding of selection-marker-free canola with *Rcr1* -rendered clubroot resistance, and has potential for developing other transgenic crops free of markers.

## Introduction

In modern agriculture, crop improvement is constantly needed to face the various challenges brought about by the increasing global population, changing climate, and the limited availability of suitable land (Tilman *et al.*, 2011). Transgenic breeding can overcome the randomness present in mutation breeding and avoid the linkage-drag and reproductive isolation found in cross-breeding, and hence it is increasingly playing a leading role in plant breeding (Chen *et al.*, 2019). However, public concerns about the potential risks of transgenic plants to human health and the environment have persisted since their first commercial use in 1992 (James, 1997). Alternative techniques such as genome editing and cisgenesis/intragenesis have been utilized for plant breeding to address concerns related to the foreign gene(s) and unwanted residue sequences (especially selection-marker genes) in transgenic plants. Genome editing can modify endogenous gene(s) for desired traits, while cisgenesis/intragenesis only use the gene-of-interest obtained from sexually compatible species (i.e. the cisgene) and the unwanted residue sequences can be removed from cis/intragenic plants (Schouten *et al.*, 2006b). Cisgenesis needs to use cisgene(s) with the native promoter and terminator, but intragenesis can use new genetic recombinations of the promoter/terminator (Rommens, 2004). To remove unwanted residue sequences in cis/intragenic plants, a promising strategy is to use sequence-specific nucleases to induce double-strand breaks flanking the sequences to be removed. The creation of two double-strand breaks on the same chromosome mainly causes deletions, and low frequencies of inversions (Pacher *et al.*, 2007; Schmidt *et al.*, 2019; Rönspies *et al.*, 2020). There are three popular sequence-specific nucleases currently in use, namely zinc-finger nucleases (ZFNs), transcription activator-like effector nucleases (TALENs), and clustered regularly interspaced short palindromic repeat (CRISPR)-associated endonucleases (Cas). Of these, CRISPR/Cas has notable advantages such as simplicity of execution, relatively low cost, high precision, and easy multiplexing, and the technique has revolutionized the field of plant genome engineering since its appearance in 2011 (Chen *et al.*, 2019; Rönspies *et al.*, 2020). Multiple studies have shown that the CRISPR/Cas system can be successfully used in plants to induce heritable deletions. For example, deletions have been induced in Arabidopsis, *Medicago truncatula*, and tomato protoplasts, obtaining deletions of up to 120 kb, 58 kb, and 3 kb, respectively, and these deletions have been successfully transferred into the germline (Čermák *et al.*, 2017; Durr *et al.*, 2018; Wu *et al.*, 2018). As the ‘second generation of transgenic crops’, cis/intragenic plants are expected to receive better public acceptance than the previous ‘traditional’ transgenic plants (Viswanath and Strauss, 2010; Lusser *et al.*, 2012; Holme *et al.*, 2013; Hou *et al.*, 2014; Dalla Costa *et al.*, 2016; Davison and Ammann, 2017; Kumar *et al.*, 2020), but they still remain quite limited in their use due to ongoing controversy over relaxing regulations on these ‘cleaner’ transgenic crops (Schouten *et al.*, 2006a; Val Giddings, 2006; Gaskell *et al.*, 2011; Ledford, 2013) and, more importantly, due to the lack of a universal workflow to develop cis/intragenic crops. Hu and Yu (2022) have reported a CRISPR/Cas9-based system that can be used to develop cisgenic germplasms in Arabidopsis; however, the system cannot be directly used for intragenic breeding, and its performance in plants other than Arabidopsis is not clear.

Clubroot, a global disease caused by the protist pathogen *Plasmodiophora brassicae*, can result in 10–15% yield losses in *Brassica* species and related crops (Dixon, 2009). *Brassica* crops include three diploid species, namely *B. rapa* (genome represented as AA; *n*=10), *B. nigra* (BB; *n*=8), and *B. oleracea* (CC; *n*=9), and three amphidiploid species, namely *B. juncea* (AABB; *n*=18), *B. napus* (AACC; *n*=19), and *B. carinata* (BBCC; *n*=17). Canola (*B. napus*; also known as oilseed rape) is an important crop as a sustainable source of healthy cooking oil and high-protein meal. In Canada, clubroot disease was first identified in canola in 2003, and since then it has quickly spread across the prairies in the provinces of Alberta, Saskatchewan, and Manitoba. Genetic resistance is deemed the most effective and economical way to control this disease (Yu *et al.*, 2021). Extensive screening of *Brassica* germplasms has been carried out over the past three decades, but only a few have been found to have clubroot resistance; for example, the European fodder turnips (*B. rapa* subsp. *rapa*) (Yu *et al.*, 2021; Yang *et al.*, 2022). Most of the resistance loci have been mapped to chromosomes A01, A02, A03, A06, and A08 of the A genome, with only a few being mapped to the B or C genome (Matsumoto *et al.*, 1998; Suwabe *et al.*, 2003, 2006; Hirai *et al.*, 2004; Piao *et al.*, 2004; Sakamoto *et al.*, 2008; Werner *et al.*, 2008; Ueno *et al.*, 2012; Chen *et al.*, 2013; Hatakeyama *et al.*, 2013; Chu *et al.*, 2014; Li *et al.*, 2016; Yu *et al.*, 2016, 2017, 2021; Huang *et al.*, 2017, 2019; Hasan and Rahman, 2018; Hirani *et al.*, 2018; Pang *et al.*, 2018; Chang *et al.*, 2019; Laila *et al.*, 2019; Fredua-Agyeman *et al.*, 2020; Karim *et al.*, 2020; Dakouri *et al.*, 2021; Rahaman *et al.*, 2022). To date, only two clubroot-resistance genes, *Crr1a* and *CRa*, have been isolated, and they both encode toll-interleukin-1 receptor, nucleotide binding site, and leucine-rich repeat (TIR-NBS-LRR, TNL) proteins (Ueno *et al.*, 2012; Hatakeyama *et al.*, 2013). *Brassica rapa* is one of the progenitor species of canola. The introgression of traits such as clubroot resistance from *B. rapa* into canola is possible via interspecific crosses; however, it takes several years to develop elite canola lines that have limited or no genetic background from *B. rapa* species such as European fodder turnips.

We have previously mapped a clubroot-resistance gene, *Rcr1*, to a refined 0.24 Mb region on chromosome A03 (Chu *et al.*, 2014) and developed several single-nucleotide polymorphism (SNP) markers that are tightly linked to the gene in *B. rapa* (Yu *et al.*, 2016). In this present study, after cloning of *Rcr1* in *B. rapa* and functional verification in canola (*B. napus*), the identity of *Rcr1* was clarified in relation to other reported clubroot-resistance genes on chromosome A03, such as *Rcr2*, *Rcr4*, and *CRa*. To demonstrate the potential of an intragenic breeding strategy, the newly identified *Rcr1* was incorporated into clubroot-susceptible elite canola lines via *Agrobacterium*-mediated transformation using a modified CRISPR/Cas9-based intragenic vector system and compared with introgression breeding using traditional hybridization and backcrossing. In less than 2 years, selection-marker-free canola germplasms with *Rcr1*-rendered stable resistant phenotypes were successfully developed within two generations, which was much faster compared to the 6 years needed for introgression. This highlights the advantages of the fast breeding strategy using the CRISPR/Cas9-based intragenic vector system compared to the traditional introgression method. It has the potential to achieve similar improvements in other crops while significantly reducing the time and labor required for breeding.

## Materials and methods

### Plants and growth conditions

The elite canola (*Brassica napus*) breeding lines DH12075 and DH16516 are both clubroot-susceptible, doubled haploid (DH), spring-type quality lines that have been developed by Dr Séguin-Swartz at the Saskatoon Research and Development Centre, Agriculture and Agri-Food Canada (SRDC, AAFC), Saskatchewan, Canada. Due to their intrinsic characteristics, DH12075 was used as the recipient plant in *Agrobacterium*-mediated transformation of *Rcr1*, while DH16516 was used for introgression breeding as the recurrent parent. Both DH lines were found to be susceptible to all the strains of *Plasmodiophora brassicae* tested in this study, so they are used as the control plants in the experiments.

Several clubroot-resistant materials were included in this study. They were the pak choy cv. ‘Flower Nabana’ (FN, *B. rapa* ssp. *chinensis*), the Chinese cabbage cv. ‘Jazz Napa Cabbage’ (JNC, *B. rapa* ssp. *pekinensis*), the turnip cultivars ECD02, ECD04, PTWG, and T19 (*B. rapa* ssp. *rapa*), the rutabaga cv. ECD10 (*B. napus* ssp. *napobrassica*), and a European oilseed rape (canola) cv. ‘Mendel’ (*B. napus*), which are all important donors for the identification of clubroot-resistance genes used in our laboratory. Resistance genes in the *Rcr1* interval have previously been found in most of them, and therefore we checked them for the presence of *Rcr1*.

The candidate gene for *Rcr1* cloned from pak choy FN was used for the intragenic breeding and in the introgression breeding as the pollen donor. For introgression breeding, briefly, pak choy FN was crossed to canola DH16516 to produce F_1_ progeny, and backcrosses with DH16516 (recurrent parent) were performed up to BC_4_. BC_4_ plants similar to the recurrent parent with resistance to clubroot were chosen to produce BC_4_S_1_ via self-pollination. Plants homozygous at the *Rcr1* locus were kept to produce the BC_4_S_2_ breeding line, designated as Y549-(0)-2-1. Marker-assisted selection with the markers SNP_A03_09 and SNP_A03_13 (Yu *et al.*, 2016) that are tightly linked to *Rcr1* was performed during the whole introgression breeding process to monitor the stabilization of the targeted clubroot-resistance locus. All plants were grown in a greenhouse under long-day conditions of 16/8 h light/dark (200 µmol m^–2^ s^–1^) at 22 °C. In experiments with heat treatments, a temperature of 37 °C was applied for 30 h after which the plants were returned to 22 °C.

### Construction of vectors

The phenotype verification of *Rcr1* was conducted using the vector pMDC32:Rcr1), which was constructed with the Gateway cloning system (Invitrogen). First, the coding sequence of *Rcr1* (4609 bp) was amplified by PCR with the primer pair caccRcr1F and Rcr1R using genomic DNA extracted from pak choy FN as the template (for primers used in this study see Supplementary Table S1). Next, the coding sequence was linked to the commercial pENTR/D-TOPO entry vector using a pENTR/D-TOPO Cloning Kit (Invitrogen). Finally, after sequence confirmation by Sanger sequencing, an LR reaction was performed to shuttle the full-length coding sequence of *Rcr1* from the pENTR/D-TOPO:Rcr1 entry vector into the Gateway destination vector pMDC32, resulting in pMDC32:Rcr1

**Fig. 1.**
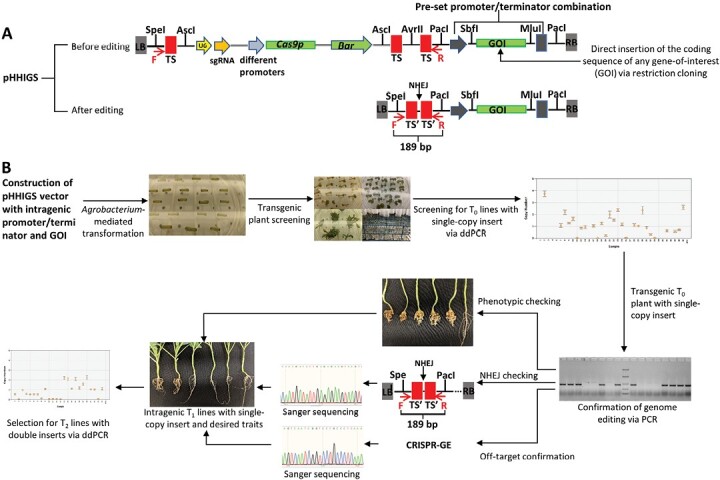
Design of the CRISPR/Cas9-based intragenic vectors and the workflow to develop intragenic germplasms. (A) pHHIGS vector design. Multiple copies of the single-guide RNA (sgRNA) targeted sequences are arranged on both sides of these to-be-removed genetic components (i.e. the sgRNA expression cassette, and the *Cas9p* and *Bar* genes) to facilitate the deletion of the flanked region, which is induced by multiple double-strand breaks happening simultaneously on the same chromosome. Since the gene-of-interest (GOI) is placed outside the flanked region, it is retained after gene editing and DNA repair via non-homologous end-joining (NHEJ). The key modification in pHHIGS vectors is the pre-set promoter/terminator linked with two unique restriction sites (i.e. SbfI and MluI) that can facilitate the direct insertion of the coding sequence of the GOI. To meet the classification requirement of intragenic crops, the promoter/terminator and the chosen GOI should all be derived from the same or sexually-compatible species as the recipient plant when constructing the custom pHHIGS vectors. The multiple restriction sites are designed to accommodate restriction cloning for vector construction. LB and RB, left and right border of the inserted T-DNA; TS, targeted sequence for the sgRNA, (i.e. a carefully designed sequence to be recognized and cut by the sgRNA/Cas9p complex), initially 23 bp including the 3 bp of the protospacer adjacent motif (PAM) sequence; TS´, a 17 bp sequence of TS remaining after editing; U6, the *U6-26* promoter from *Arabidopsis thaliana*; sgRNA, the DNA template and scaffold sequences for sgRNA transcription; *Cas9p*, the *Cas9* gene modified with plant-optimized codons; *Bar*, phosphinothricin acetyltransferase. (B) A streamlined workflow to develop intragenic crops using the pHHIGS vectors. ddPCR, droplet digital PCR.

For the demonstration of intragenic breeding in this study, a cisgenic vector system was modified and utilized (Fig. 1A). In our previous study, a series of CRISPR/Cas9-based vectors were constructed to facilitate cisgenic plant breeding, namely pHHCGS-AP1, pHHCGS-CLV3, and pHHCGS-Hsp18.2 (where ‘CG’ represents ‘CisGenesis’; Hu and Yu, 2022). The different promoters in these vectors provided more control over the expression of *Cas9p*. The promoters *CLV3* and *AP1*, derived from the Arabidopsis genes *CLAVATA3* (‘early stem cell identity’) and *APETALA1* (‘flower meristem identity’), could be induced by developmental cues at an early and middle growth stage, respectively (Van Ex *et al.*, 2009), while the promoter *Hsp18.2* could be activated by 2 h of heat treatment at 37 °C (Takahashi *et al.*, 1992) (see Supplementary Fig. S1 for the full sequences of the different promoters). Regarding the auto-excision feature of the cisgenic vectors, briefly, multiple copies of the CRISPR/Cas9-targeted sequences were placed on both sides of the components to be removed, i.e. the unwanted residue sequences in the final products including the selection marker gene (*Bar*), the single-guide RNA (sgRNA) expression cassette driven by the promoter *AtU6-26*, and the *Cas9p* gene driven by the different promoters, whilst the gene-of-interest was placed outside the flanked region. Thus, after successful gene editing, the unwanted residue sequences could be deleted from the final products while the desired gene-of-interest would remain. Due to the intrinsic requirements of cisgenic breeding, only an intact gene-of-interest including its original promoter/terminator could be used with the pHHCGS vectors. In contrast, recombinant regulatory elements such as the promoter/terminator could be used for intragenic breeding, which offers more flexibility for breeders especially when dealing with a gene-of-interest that is not yet fully characterized. Hence, we modified the cisgenic vectors by adding a pre-set promoter/terminator combination linked with two unique restriction enzyme sites (SbfI and MluI) at site PacI of the three pHHCGS vectors, resulting in three vectors with different promoters (i.e. pHHIGS-AP1, pHHIGS-CLV3, and pHHIGS-Hsp18.2, where ‘IG’ represents ‘IntraGenesis’) (Fig. 1A). This modification kept the core design of the entire flanked region in the pHHCGS vectors whilst facilitating direct insertion of the coding sequence of any gene-of-interest for intragenic breeding. In this study, the maize ubiquitin promoter (*pubi*) and the NOS terminator (*tnos*) were amplified from pYLCRISPR/Cas9Pubi-B (Ma *et al.*, 2015) and utilized in our pHHIGS vectors. *pubi* was selected because it is a plant-origin promoter that has been widely used to constitutively drive transgene expression in both monocot and dicot plants, and the driving activity is strong, especially in rapidly dividing cells (Cornejo *et al.*, 1993; Christensen and Quail, 1996; Ma *et al.*, 2015). Breeders could choose other promoters/terminators to replace our choice via restriction cloning to build their customized pHHIGS vectors, but the promoter/terminator of choice should be derived from the same recipient plant or from sexually compatible relatives to meet the classification requirement of intragenic crops. To construct the expression vector pHHIGS:Rcr1, the full-length coding sequence of *Rcr1* was amplified with the primer pair SbfI-Rcr1F and MluI-Rcr1R, and directionally inserted into the pHHIGS vectors digested by SbfI/MluI (Supplementary Table S1). Three versions of pHHIGS:Rcr1 with different promoters (*AP1*, *CLV3*, and *Hsp18.2*) were constructed and tested for their performance in intragenic plant breeding in canola.

### Transformation

The constructs were delivered into *Agrobacterium tumefaciens* GV3101 individually via electroporation. For the transformation and regeneration of DH12075, the procedure described by Cardoza and Stewart (2003) was followed, and the antibiotic hygromycin B or the herbicide Basta were used in the screening process for positive transformants transformed by the pMDC32 or pHHIGS vectors, respectively. After being transferred to soil, the rooted shoots were grown to maturity under long-day conditions. Positive transformants (T_0_) were confirmed with PCR and/or droplet digital PCR (ddPCR), and seeds of positive T_0_ lines were collected and used in the various experiments.

### PCR tests

Total DNA was extracted from the young leaves of each plant with DNeasy Plant Mini Kits (Qiagen), and used as the template in regular PCR and ddPCR. PCR was used to clone *Rcr1* from the various clubroot-resistant materials and to check for the presence of various genes in transformed plants, namely the gene-of-interest (*Rcr1*), the selection marker gene (*Bar*), and the CRISPR/Cas9 components (*Cas9p*). ddPCR was used to evaluate the copy number of inserted T-DNA in positive canola plants. Quantitative reverse-transcription PCR (RT-qPCR) was used to assess the relative expression levels of several target genes, such as *Rcr1* and *Cas9p*. For *Rcr1*, total RNA was extracted from the roots of each plant (at least three plants of each disease grade) at 10 d post-inoculation using RNeasy Plant Mini Kits (Qiagen), while for *Cas9p* and sgRNA various plant tissues (such as young leaves and flowers in different experiments) were collected from each plant (at least three plants of each treatment) and used for RNA extraction. The reaction conditions of each PCR system were the same as described by Hu and Yu (2022). The *B. napus* housekeeping gene *BnACTIN2* was utilized as a reference gene in the data analysis of ddPCR and RT-qPCR. For ddPCR, the copy number of insertions was calculated based on the ratio of genomic copies of the target gene (*Rcr1* or *Bar*) versus *BnACTIN2*. For RT-qPCR, the expression level of *BnACTIN2* was found to be stable in all the RNA samples tested (threshold cycle values ~22.3) and thus it was used as an internal control to normalize the quantitative results of the relevant target genes. For transgenic lines obtained with *Agrobacterium*-mediated transformation, three individual T_0_ lines with single-copy insert of each vector were used as biological replicates, and at least three progeny plants from each replicate were used in the various tests and data analysis. All PCR primers used in this study are listed in Supplementary Table S1.

### Characterization of editing activities in canola controlled by different promoters and heat treatments

The editing activity controlled by the promoters of *AP1* and *CLV3* could occur naturally with growth, while the promoter of *Hsp18.2* needs heat induction. To check the tissue-specific expression pattern of *Cas9p* controlled by the three promoters, four types of tissues (young leaf, flower, root, and stem) were collected when flowering began. All plants were grown under normal long-day conditions, while an additional set of tissue samples were collected from plants with pHHIGS-Hsp18.2 that had received a heat treatment to activate the *Hsp18.2* promoter. To check for synergetic effects of heat treatments and different promoters, the experimental design and data analysis described by Hu and Yu (2022) was followed, with minor modifications. Briefly, three T_0_ lines with a single-copy insert, serving as three biological replicates, were selected for each vector, and 10 T_1_ plants of each T_0_ line received one of the four following treatments: HT1, only one heat treatment (37 °C for 30 h) after 7 d of acclimation in soil; HT2, only one heat treatment before entering the reproductive phase, ~28 d after HT1; HT1 + 2, combination of the two heat treatments; No HT, normal long-day conditions throughout the experimental period. Newly expanded leaves were collected from each plant at four sampling points: L1, before HT1; L2, 42 h after HT1; L3, before HT2; and L4, 42 h after HT2. The tissue samples were used to isolate total DNA and total RNA, which were tested by PCR (for presence or absence of the target gene) and RT-qPCR (for monitoring gene expression levels), respectively. The editing efficiency was determined by dividing the total number of edited plants by the total number of positive plants. A positive plant was defined as one in which the presence of the target gene (e.g. *Bar*) was detected by PCR in L1 leaves, and an edited plant was identified when a positive plant (initially confirmed by PCR in L1) subsequently exhibited absence of the target gene as determined by PCR in L4 leaves.

### Pathogenicity tests

Clubroot pathogenicity tests were performed as described by Yu *et al.* (2017). Briefly, the root systems of 1-week-old seedlings were dipped in a resting spore suspension, and the seedlings were then planted individually in pots containing potting mixture. After 6 weeks, the roots were examined for club formation. For canola plants transformed by the pMDC32:Rcr1 or pHHIGS vectors, the seeds of T_0_ lines carrying a single-copy insert and their progenies were used in a series of tests. For the introgression process, the tests were used in conjunction with marker-assisted selection throughout the generations to monitor the stabilization of the *Rcr1* locus. Three strains of the clubroot pathogen *P. brassicae* collected in western Canada, namely 3H (Alberta), SK25 (Saskatchewan), and PSI11 (Manitoba), were kindly provided by Dr Stephen Strelkov (University of Alberta), Dr Alireza Akhavan (Government of Saskatchewan), and Dr Lee Anne Murphy and Mr Xiaowei Guo (Pest Surveillance Initiative, Winnipeg, Manitoba), respectively. These strains were selected because they possibly carry an avirulence gene that interacts with the resistance gene *Rcr1*. Disease rating was performed using a 0–3 scale at 6 weeks after inoculation (Kuginuki *et al.*, 1999), and an improved method was used to calculate the disease severity index (DSI; Strelkov *et al.*, 2006). The canola recipient line DH12075 for transformation and the elite parental line DH16516 for introgression were included as susceptible controls in the pathogenicity tests.

### Sanger sequencing

Sanger sequencing was used in conjunction with PCR to confirm gene-editing events and possible off-target mutations. After PCR, amplicons were cloned into the commercial pCR 2.1 vector using a TA Cloning Kit (Invitrogen). For plants transformed by pHHIGS-AP1, pHHIGS-CLV3, or pHHIGS-Hsp18.2, three T_0_ canola plants and their progenies were selected for sequencing based on their screening for single-copy insertion and successful editing results. In the screening of off-target mutations, for each combination of vector and the potential target site, at least three clones were used for Sanger sequencing.

### Statistical analysis

One-way ANOVA with post-hoc Tukey’s HSD tests was performed to determine the significance of results (*P*<0.05).

## Results

### *Rcr1* cloning and functional verification

Our previous fine-mapping study using an F_1_ population constructed from a cross between the resistant pak choy cv. ‘Flower Nabana’ (FN; *B. rapa* ssp. *chinensis*) and a susceptible canola *B. rapa* DH line ACDC located four TNL-type disease-resistance genes on chromosome A03 of the Chinese cabbage ‘Chiifu’ reference genome (V1.2; http://brassicadb.cn), namely *Bra019409*, *Bra019410*, *Bra019412*, and *Bra019413* (Chu *et al.*, 2014). However, with primers designed based on these four candidates, none of the cloned genes from pak choy FN showed a clubroot-resistance phenotype when transformed into the susceptible canola line DH12075. After further examination of the sequences of the cloned genes, the PCR product of *Bra01940*9 (4619 bp) from pak choy FN was found to share high similarity (99.87%) with the previously isolated *CRa* (4609 bp) from Chinese cabbage T136-8 (*B. rapa* ssp. *pekinensis*) (Ueno *et al.*, 2012), except for several base differences at both ends (i.e. the locations of both primers; see sequence alignment in Supplementary Fig. S2). This prompted us to redesign the primer pair, and this resulted in a new candidate *Rcr1* gene being cloned from pak choy FN, which was identical to *CRa* in the coding region.

**Fig. 2.**
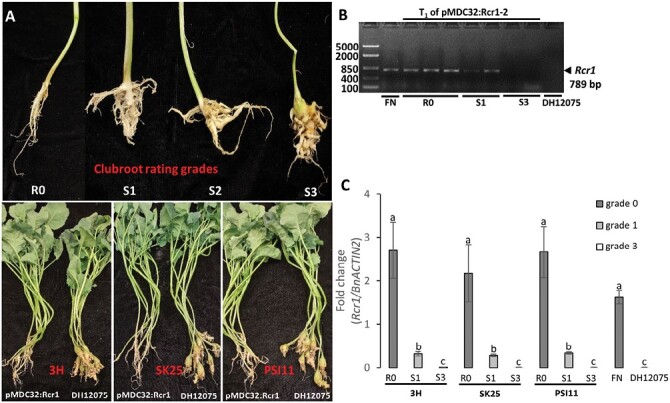
Functional verification of *Rcr1*. The full-length genomic coding sequence of *Rcr1* was cloned from the clubroot-resistant pak choy cv. ‘Flower Nabana’ (FN, *Brassica rapa* ssp. *chinesis*) and inserted into the plant binary vector pMDC32. The Gateway expression vector pMDC32:Rcr1 used the constitutive promoter of CaMV 35S to drive *Rcr1* expression in the elite canola cv. DH12075 (*B. napus*), which is susceptible to all the clubroot strains used in this study. Thus, FN and DH12075 were used as the positive and susceptible control, respectively. (A) Disease phenotyping. The top image shows the scale of disease severity, as assessed at 6 weeks after inoculation: R0, resistant plants; S1, slightly susceptible; S2, moderately susceptible; and S3, fully susceptible. The bottom image shows comparisons between the transformed plants and the susceptible control for each of the *Plasmodiophora brassicae* strains used in this study: 3H from Alberta; SK25 from Saskatchewan; and PSI11 from Manitoba. The plants illustrating the disease severity scale were infected by the 3H strain. (B) PCR genotyping of resistant and susceptible T_1_ plants infected by the 3H strain. (C) Relative expression of *Rcr1* in resistant and susceptible T_1_ plants as determined by RT-qPCR, with the data normalized to the expression of *BnACTIN2*. Data are means (±SD) of three replicates. Different letters indicate significant differences as determined using one-way ANOVA with post-hoc Tukey HSD tests (*P*=0.05).

For functional analysis of the candidate *Rcr1*, a series of clubroot pathogenicity tests were carried out on susceptible canola DH12075 transformed with pMDC32:Rcr1. The results showed that the T_1_ populations of three independent *Rcr1*-overexpression T_0_ lines (pMDC32:Rcr1-2, pMDC32:Rcr1-5, and pMDC32:Rcr1-6) showed the same resistance spectrum as the source plant pak choy FN, for the three *P. brassicae* strains used in this study (3H, SK25, and PSI11; Fig. 2A). The T_1_ plants were also checked for the presence of *Rcr1* and for its relative expression (Fig. 2B, C). Rarely, slightly susceptible plants (graded as S1; ~0–3% of the total number of plants) were identified and tested positive for the *Rcr1* gene, but the fully susceptible plants (S3; ~22–27% of the total) all tested negative (Fig. 2B). In addition, the expression level of *Rcr1* in resistant plants (designated R0; ~72–77% of the total) was significantly higher than in susceptible ones, indicating that expression of *Rcr1* affected the resistance phenotype (Fig. 2C). These results suggested that the cloned *Rcr1* is a functional clubroot-resistance gene.

To check for the presence of *Rcr1* in the clubroot-resistant materials and to examine the relationships of the various clubroot-resistance loci mapped in the close region of *Rcr1*, such as *Rcr2* (Huang *et al.*, 2017), *Rcr4* (Yu *et al.*, 2017), *Rcr5* (Huang *et al.*, 2019), and the clubroot-resistance gene on A03 in the oilseed rape cultivar ‘Mendel’ (*B. napus*), gene cloning with the *Rcr1* primers (Supplementary Table S1) was performed on an extended list of *B. rapa* and *B. napus* breeding cultivars and our own in-house breeding lines. Sequencing results showed that most of the clubroot-resistant materials tested carried this resistance gene (Table 1), indicating a common clubroot-resistance source on the A genome of these *B. rapa* (AA) and *B. napus* (AACC) materials.

**Table 1.** Summary of *Rcr1* presence in popular *Brassica* crops and our in-house canola breeding lines

Plant group	Cultivar	Source of clubroot resistance	Reported clubroot-resistance locus	Sequence similarity with *Rcr1*
Pak choy (*Brassica rapa* subsp. *chinensis*, AA genome)	Flower Nabana (FN)	FN	*Rcr1* (Chu *et al.*, 2014)	100%
Chinese cabbage (*B. rapa* ssp. *pekinensis*, AA genome)	Jazz Napa Cabbage (JNC)	JNC	*Rcr2* (Huang *et al.*, 2017)	100%
Turnip (*B. rapa* ssp. *rapa*, AA genome)	ECD02	ECD02	*CRa* (Ueno *et al.*, 2012)	100%
	ECD04	ECD04	*CRA3.7* (Yang *et al.*, 2022)	100%
	Purple Top White Globe (PTWG)	PTWG	*Rcr5* (Huang *et al.*, 2019)	N/A
	T19	T19	*Rcr4* (Yu *et al.*, 2017)	100%
Rutabaga (*B. napus* ssp. *napobrassica*, AACC genome)	ECD10	ECD10	–	N/A
Canola (*B. napus* L., AACC genome)	Mendel	ECD04	A clubroot-resistance gene on A03 of ECD04	100%
	Y30-SK-002	Mendel	A clubroot-resistance gene on A03 of ECD04	100%
	Y549-(0)-2-1	FN	*Rcr1* (Chu *et al.*, 2014)	100%
	DH12075	(None)	–	N/A
	DH16516	(None)	–	N/A

N/A, no amplification of *Rcr1* despite multiple attempts. –, no clubroot-resistance gene reported or no clubroot resistance identified at the *Rcr1* locus. DH12075 and DH16516 are susceptible cultivars.

### Successful development of selection-marker-free canola with clubroot resistance using the modified pHHIGS vectors

**Fig. 3.**
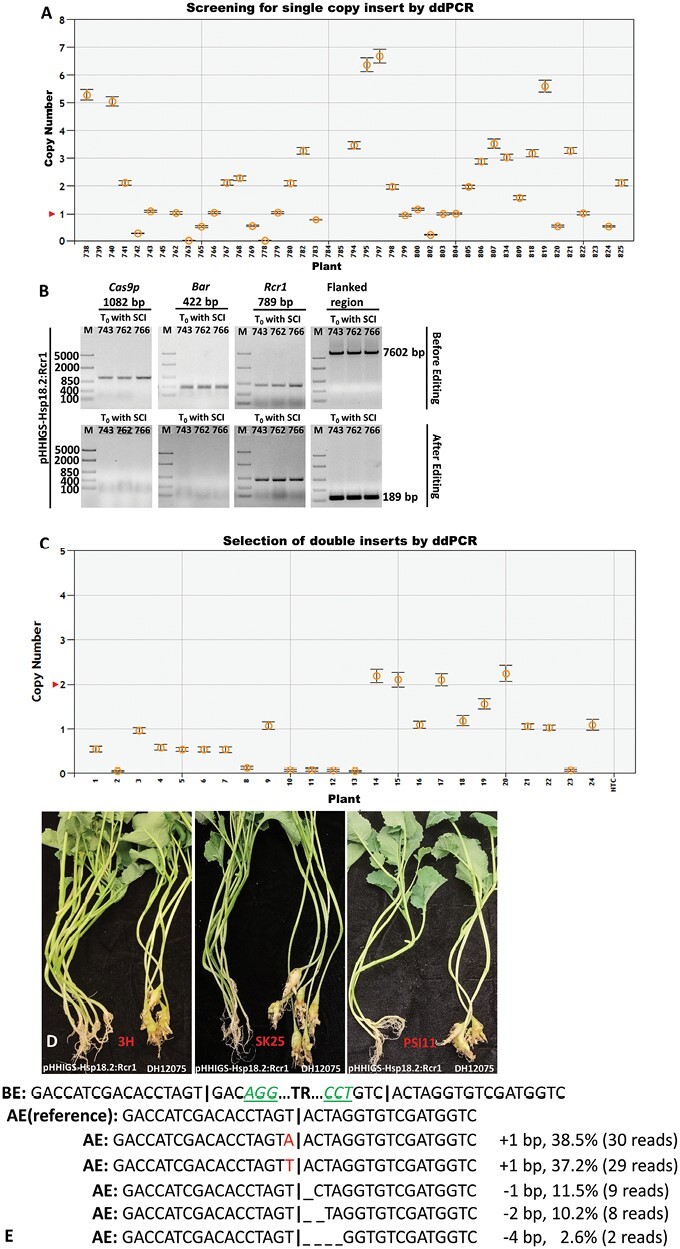
Demonstration of successful development of selection-marker-free canola with clubroot resistance using the pHHIGS vector system. The three pHHIGS constructs under the control of different promoters (i.e. pHHIGS-AP1:Rcr1, pHHIGS-CLV3:Rcr1, and pHHIGS-Hsp18.2:Rcr1) were used to develop selection-marker-free canola with clubroot resistance rendered by *Rcr1*. Similar results were obtained for three constructs throughout the entire workflow from genotyping by ddPCR/PCR to phenotyping by pathogenicity tests, and hence only the results using pHHIGS-Hsp18.2:Rcr1 are presented as a representative example. (A) Droplet digital PCR (ddPCR) screening for T_0_ lines with a single-copy insert of the T-DNA. Three such lines (743, 762, and 766) and their progenies were selected and subject to various genotyping and phenotyping tests, and served as three biological replicates. (B) PCR results for the three selected T_0_ lines showing distinct differences for *Cas9p* (1082 bp), *Bar* (422 bp), and *Rcr1* (789 bp) in the same plant before and after editing. The PCR results also confirmed that the entire flanked region (a total of 7602 bp in pHHIGS-Hsp18.2) was removed as designed, leaving a remnant scar sequence of 189 bp after successful editing and DNA-repair via non-homologous end-joining (NHEJ). (C) ddPCR screening for T_1_ lines with double inserts of *Rcr1*. (D) Resistance phenotypes in the T_2_ population of a selected T_1_ line with double inserts of *Rcr1* (line 15), compared with the untransformed control, DH12075. The three *Plasmodiophora brassicae* strains were collected from the Alberta (3H), Saskatchewan (SK25), and Manitoba (PSI11) provinces of Canada. (E) Sanger sequencing results showing the joint sequence after editing and repair via NHEJ. BE, before editing; AE, after editing; TR, truncated region. The cutting site is indicated by **|**.

With the modified pHHIGS vectors, the 4609 bp coding sequence of *Rcr1* was cloned using the primers SbfI-Rcr1F and MluI-Rcr1R, and directly inserted into the pre-set location immediately after the constitutively expressed maize *ubiquitin* promoter. We constructed three vectors, pHHIGS-AP1, pHHIGS-CLV3, and pHHIGS-Hsp18.2, which differed only at the promoters controlling the expression of *Cas9p*, and used them to transform the susceptible canola breeding line DH12075 via *Agrobacterium* (Fig. 1A). The original T_0_ transgenic canola plants were screened by ddPCR for single-copy insertion of T-DNA, and 9, 7, and 10 plants were identified to carry single-copy inserts of pHHIGS-AP1, pHHIGS-CLV3, and pHHIGS-Hsp18.2, respectively (Fig. 3A; similar results from genotyping to phenotyping were found for all three vectors, so the figure shows only pHHIGS-Hsp18.2 as a representative example). Checking by PCR of the three target genes *Bar*, *Cas9p*, and *Rcr1* showed that they all presented in the positive transformants before editing; in contrast, after editing the unwanted residue genes *Bar* and *Cas9p* were no longer detected while *Rcr1* remained. PCR also confirmed that the entire flanked region (a total of 7602 bp for pHHIGS-Hsp18.2) was removed as designed, leaving a remnant scar sequence of 189 bp after successful editing and DNA repair via non-homologous end-joining (NHEJ; Fig. 3B). For phenotyping of disease resistance, only progenies of those T_0_ lines carrying the single-copy insert were used in the pathogenicity tests because the original T_0_ plants were generated from tissue culture and were not suitable for the tests. In the T_1_ populations, the copy number of inserts generally followed Mendel’s law of segregation, namely a ratio of 1:2:1 for two copies, one copy, and zero copies (Fig. 3C), and a similar agreement with the rule was also observed in T_2_ populations (Table 2). Pathogenicity tests showed that the T_1_ populations all exhibited strong resistance to *P. brassicae* (DSI=24.1% or lower), and the T_2_ populations showed complete resistance when the progenies of T_1_ lines with two copies of inserts were tested (Table 2; Fig. 3D). A further examination of the T_2_ plants of three selected T_1_ plants harboring two copies of *Rcr1* showed that all the progenies also had two copies of *Rcr1*, which indicated that these T_1_ plants were homozygous at the *Rcr1* locus, and thus it could be stably passed on to future generations. The excellent performance in disease resistance and the significant difference compared with untransformed susceptible DH12075 plants (DSI=100%) could be attributed to the presence of at least one copy of *Rcr1* in the majority of the populations (75% or more) (Table 2). To confirm the editing results, Sanger sequencing was performed on the PCR products of the truncated region (i.e. the 189 bp of the remnant scar sequence) using a set of primers from both borders of the T-DNA (Supplementary Table S1; see primers ‘F’ and ‘R’ in Fig. 1A). As shown in Fig. 3E, five types of minor mutations were identified at the joining site.

**Table 2.** Distribution of copy numbers of *Rcr1* and disease severity index (DSI) in the T_0_–T_2_ generations of canola lines transformed with the different vectors

Vector	T_0_	T_1_ of T_0_ with single-copy insert					T_2_ of T_1_ with two copies				
	1 copy	2 copies	1 copy	0 copy	Total no. plants	DSI (%)	2 copies	1 copy	0 copy	Total no. plants	DSI (%)
pHHIGS-AP1	9	15	32	13	60	21.7 ± 5.8^b^	59	0	0	59	0 ± 0^a^
pHHIGS-CLV3	7	14	30	14	58	24.1 ± 1.6^b^	60	0	0	60	0 ± 0^a^
pHHIGS-Hsp18.2	10	14	33	13	60	21.7 ± 2.9^b^	60	0	0	60	0 ± 0^a^
DH12075	–	0	0	60	60	100 ± 0^c^	0	0	60	60	100 ± 0^c^

Copy number was determined by ddPCR; DSI was determined through pathogenicity tests. Different letters indicate significant differences in DSI within the T_1_ and T_2_ generations, as determined using ANOVA and post-hoc Tukey’s HSD test.

### Heat treatment significantly improves the performance of intragenic vectors in canola

We next examined the expression levels of CRISPR/Cas9 components (*Cas9p*) in different tissues in plants transformed with the three vectors (Table 3). Although variations in *Cas9p* expression levels in the different tissues were observed (from undetectable to 1.6-fold compared to *BnACTIN2*), moderate-to-high levels of expression were consistently identified in the flower tissue (heat treatment required for plants carrying pHHIGS-Hsp18.2). The expression pattern that we observed was in agreement with previously reported expression patterns of *AP1*, *CLV3*, and *Hsp18.2* in Arabidopsis (Alejandra Mandel *et al.*, 1992; Fletcher *et al.*, 1999; Takahashi *et al.*, 1992; Van Ex *et al.*, 2009), and it also further confirmed that the deletion of unwanted residue sequences in the current generation could transfer to the progenies through germline cells (i.e. heritable deletion) (Van Ex *et al.*, 2009).

**Table 3.** Relative expression of *Cas9* under the control of the different promoters in various tissues of transformed canola lines

Vector	Leaf	Flower	Root	Stem
pHHIGS-AP1	0.44 ± 0.045^a^	1.0 ± 0.058^c^	–	–
pHHIGS-CLV3	0.70 ± 0.092^b^	1.1 ± 0.044^c^	–	0.67 ± 0.050^b^
pHHIGS-Hsp18.2 (no heat treatment)	–	–	–	–
pHHIGS-Hsp18.2 (with heat treatment)	1.6 ± 0.055^d^	1.6 ± 0.046^d^	0.65 ± 0.058^b^	1.5 ± 0.029^d^

Relative expression of *Cas9p* is shown as the fold-change measured by RT-qPCR with the data normalized to the expression of *BnACTIN2*; –, no expression detected. For pHHIGS-Hsp18.2, a heat treatment of 37 °C was applied for 30 h immediately after the first batch of flowers bloomed.

**Fig. 4.**
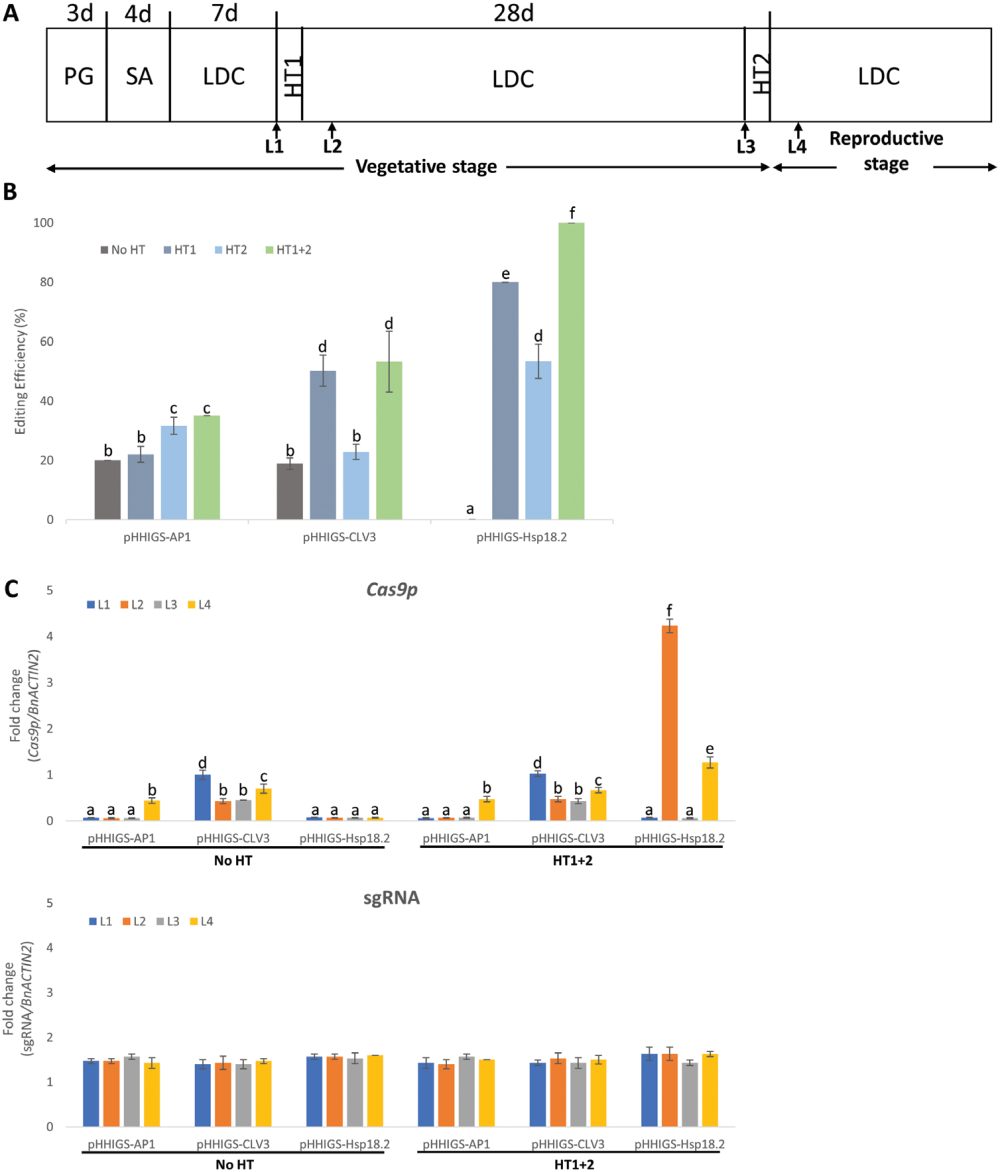
Heat treatments significantly improved the performance of the intragenic pHHIGS vectors in canola. (A) Schematic diagram of the time-course (in days) of experiments involving heat treatment (HT). PG, pre-germination period on plates; SA, soil acclimation period; LDC, long-day conditions (16/8 h light/dark at 22 °C); HT1 and HT2, first and second heat treatments, each at 37 °C for 30 h. L1–L4 indicate the sampling of newly expanded leaves; L1 and L3 were sampled immediately before HT1 and HT2, respectively, whilst L2 and L4 were sampled at 42 h after HT1 and HT2, respectively. (B) Editing efficiencies under the different heat treatments and promoters. (C) Relative expression levels of *Cas9p* and sgRNA in leaves of plants under different heat treatments and promoters, as determined by RT-qPCR with the data normalized to the expression of *BnACTIN2*. Data are means (±SD) of three replicates. Different letters indicate significant differences as determined using one-way ANOVA and post-hoc Tukey’s HSD tests (*P*=0.05).

We then applied a series of heat treatments to the canola plants transformed with the different intragenic vectors (Fig. 4A). Without heat treatment ( ‘No HT’), a moderate percentage of plants (~18.9–20.0%) transformed with pHHIGS-CLV3 and pHHIGS-AP1 were edited, but no plants transformed with pHHIGS-Hsp18.2 were edited, which might indicate the basal performance of these promoters without heat-treatment stimulation. When heat treatments were applied ( ‘HT1’, ‘HT2’, or ‘HT1 + 2’; Fig. 4A), plants transformed with pHHIGS-CLV3 showed significantly higher editing efficiency (50.2–53.2%) in ‘HT1’ and ‘HT1 + 2’ than in ‘HT2’ (22.8%; Fig. 4B), whilst for plants transformed with pHHIGS-AP1, ‘HT2’ and ‘HT1 + 2’ significantly improved the editing efficiency (31.7–35.0%) compared with ‘HT1’ (22.0%). The editing efficiency in plants transformed by pHHIGS-Hsp18.2 showed significant increases from ‘HT2’ (53.3%) to ‘HT1’ (80.0%), and complete editing occurred in ‘HT1 + 2’ (100%). As observed previously in Arabidopsis with cisgenic vectors, the heat treatments showed similar and significant impacts on editing efficiencies of all three intragenic vectors in canola, which could be due to the synergetic effect between heat treatments and the sgRNA/Cas9p complex (Hu and Yu, 2022). Since the ‘HT1 + 2’ treatment showed the most consistent and significant impact on editing efficiency for plants transformed by all three intragenic vectors, we used it to further examine the expression of the sgRNA/Cas9p complex in comparison with ‘No HT’.

We found that sgRNA showed a constant level of expression across all vectors and both heat treatments (1.47- to 1.63-fold that of *BnACTIN2*) (Fig. 4C), which indicated that a sufficient amount of sgRNA was present in all the cases studied. In contrast, distinct variations in the expression levels of *Cas9p* were observed among the different vectors and heat treatments. Across both heat treatments, when driven by the *CLV3* promoter, the expression level of *Cas9p* dropped from being at about the same as *BnACTIN2* at L1 to 0.45-fold at L2 and L3, and 0.70-fold at L4; while for the *AP1* promoter, the expression of *Cas9p* showed a sudden increase from undetectable levels at L1–L3 to 0.44-fold that of *BnACTIN2* at L4. The lack of difference between ‘H1 + 2’ and ‘No HT’ for these two promoters indicated that the increased enzyme activity of Cas9p at 37 °C (LeBlanc *et al.*, 2018) might be responsible for the significantly higher editing efficiency observed in ‘HT1 + 2’ (Fig. 4B). The expression of *Cas9p* can be fully activated by heat treatments when driven by the *Hsp18.2* promoter (Takahashi *et al.*, 1992), and this is consistent with a peak level of *Cas9p* expression (4.2-fold that of *BnACTIN2*) being observed immediately after the first heat treatment (L2; Fig. 4C), and the complete editing seen in the canola plants transformed with pHHIGS-Hsp18.2 (Fig. 4B) is likely to be attributable to the high levels of *Cas9p* expression detected from L2 to L4.

### No detectable off-target mutations in CRISPR/Cas9-edited canola

Some studies have reported low-frequency off-target mutations in plants when using CRISPR/Cas9 for genome editing, and there is an ongoing discussion regarding whether this needs to be fully rectified before the technique can be used for crop improvement (Zhang *et al.*, 2016; Chen *et al.*, 2019). We have addressed this concern in the design of the pHHCGS vectors, with much deliberation involved in choosing the best possible target sequence for sgRNA (Hu and Yu, 2022); however, prediction using CRISPR-GE (http://skl.scau.edu.cn/) (Xie *et al.*, 2017) indicated that potential off-target loci with low off-target scores still existed in the canola (*B. napus*) background. Because the protospacer-adjacent motif (PAM) and the ‘seed sequence’ (12 nucleotides adjoining the PAM) are critical for the recognition specificity of Cas9 nuclease (Doench *et al.*, 2016), we selected 11 putative off-target sites for canola based on these critical sequences and the predicted off-target scores, and examined them by PCR and Sanger sequencing. The sequencing results showed no mutations at the predicted off-target sites (Table 4), which indicated that the gene-editing activities induced by the intragenic vectors were highly specific in the canola background.

**Table 4.** Results of Sanger sequencing for possible off-target mutations

Chromosome	Position	Sequence^1^	Off-target score^2^	Gene/Region^3^	Number of mutations
A04	15477816	GAC**T**AT**G**GACAC**T**TAGTG**T**C **TGG**	0.142	Intergenic	0
C09	17393743	GACCATC**T**ACA**A**CT**G**GTGA**TAGG**	0.087	Intergenic	0
C04	32957300	GACCAT**A**GA**G**ACCTAGT**CT**C **CGG**	0.075	*BnaC04g31070D*/CDS	0
A06	13311576	GACCATC**A**AC**G**C**AC**AGTGAC **AGT**	0.001	Intergenic	0
C09	26445152	GACCA**C**C**T**ACA**T**C**G**AGTGAC** AGG**	0	Intergenic	0
C08	28724650	**T**ACCATCGAC**G**CC**C**A**C**TGAC **AGT**	0	*BnaC08g27880D*/CDS	0
A09	26310248	**T**ACCATCGAC**C**CC**C**A**C**TGAC **AGT**	0	*BnaA09g36320D*/CDS	0
C09	1696913	GACCATCGAC**C**CC**A**A**C**T**AC**C** AGG**	0	Intergenic	0
A05	8038516	GAC**T**ATCGACAC**T**T**CT**TGAC** ACG**	0	Intergenic	0
C01	1462760	GA**T**CATC**A**ACACCTA**CAA**AC** AGG**	0	*BnaC01g42300D*/CDS	0
A01	18409107	G**T**CCATC**A**ACACC**G**AG**A**GAC** ACG**	0	*BnaA01g26340D*/CDS	0

^1^The target sequence recognized by our designed sgRNA was GACCATCGACACCTAGTGAC. In the predicted off-target sites, the PAM sequence (NGG) is highlighted in green, and mismatched bases are indicated in red.

^2^Off-target scores were obtained from CRISPR-GE (http://skl.scau.edu.cn/).

^3^ *Brassica napus* reference genome Genoscope (V5) cited from CRISPR-GE (http://skl.scau.edu.cn/).

### Comparison between intragenic and introgression breeding for clubroot resistance in canola

**Fig. 5.**
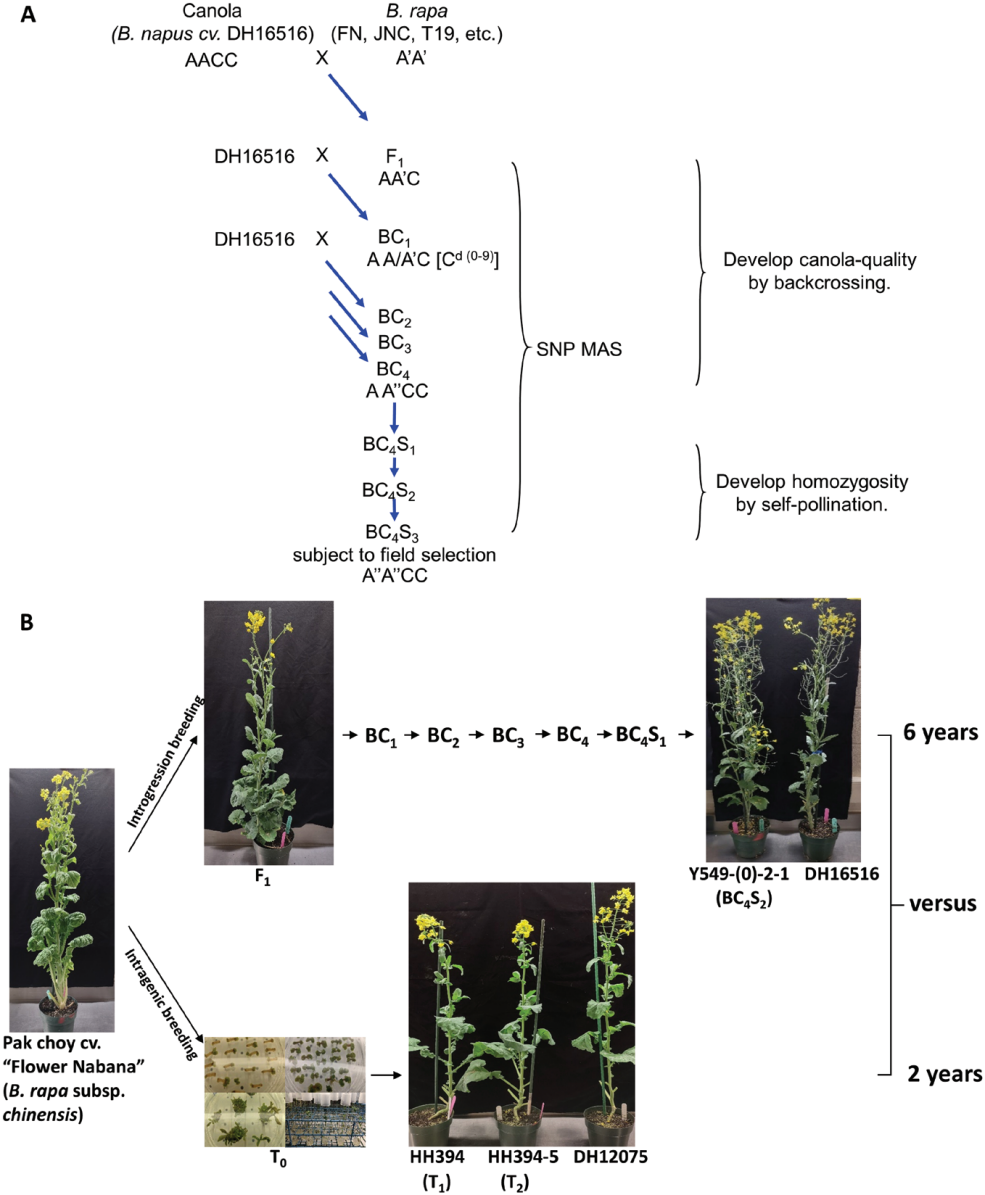
Comparison of the breeding process between intragenic and introgression canola with resistance to clubroot (*Plasmodiophora brassicae*) rendered by *Rcr1*. (A) Flowchart showing the introgression of a *Brassica rapa* clubroot-resistance gene into *B. napus* spring-type canola. FN, pak choy cv. ‘Flower Nabana’ (*B. rapa* subsp*. chinensis*); JNC, Chinese cabbage cv. ‘Jazz Napa Cabbage’ (*B. rapa* ssp. *pekinensis*); T19, Turnip cv. ‘T19’ (*B. rapa* ssp. *rapa*); DH16516, clubroot-susceptible canola cv. ‘DH16516’ (*B. napus*); SNP, single-nucleotide polymorphism; MAS, marker-assisted selection. A and A´ indicate the A genome from different sources, where A carries no clubroot-resistance gene and A´ does. A´´ is the new A genome after homologous recombination between A and A´ and it carries the clubroot-resistance gene. (B) Comparison of the breeding process between conventional introgression and intragenic breeding. DH12075 is another clubroot-susceptible canola cultivar.

In this study, we successfully developed multiple canola germplasm lines showing *Rcr1*-rendered clubroot resistance through two different routes. One route was traditional introgression and the other used an intragenic method. As shown in Table 5, the new germplasms developed via the different routes had similar levels of clubroot resistance to the three strains of *P. brassicae* collected in western Canada. Intriguingly, the selection-marker-free canola with a stable clubroot-resistance phenotype could be developed within two generations using the modified pHHIGS vector system and workflow (Fig. 1), whereas the traditional introgression process needed at least eight generations to achieve the same outcome (Fig. 5A). This represents an almost three-fold difference in breeding time for canola (~2 years for intragenic breeding versus 6 years for introgression breeding; Fig. 5B). Furthermore, introgression has an intrinsic requirement for compatibility in cross-breeding but some relative species (especially wild ones) are recalcitrant against crossing, and hence this route might not be a viable option to incorporate valuable traits identified in such species.

**Table 5.** Scores for disease severity index (DSI, %) for canola germplasms developed by either intragenic or introgression breeding

			Pathogen strain		
	Germplasm	Generation	3H (Alberta)	SK25 (Saskatchewan)	PSI11 (Manitoba)
	Pak choy cv. ‘Flower Nabana’ (*Brassica rapa* ssp. *chinesis*)	Clubroot-resistance donor	0 ± 0^c^	0 ± 0^c^	0 ± 0^c^
Intragenic breeding	Canola cv. DH12075 (*B. napus*)	Recipient line	100 ± 0^a^	92.6 ± 12.8^a^	100 ± 0^a^
	HH394	T_1_	24.1 ± 1.6^b^	23.3 ± 2.9^b^	22.4 ± 2.5^b^
	HH394-5	T_2_	0 ± 0^c^	0 ± 0^c^	0 ± 0^c^
Introgression breeding	Canola cv. DH16516 (*B. napus*)	Recurrent parent	97.2 ± 4.8^a^	96.3 ± 6.4^a^	100 ± 0^a^
	Y549-(0)-2-1	BC_4_S_2_	2.8 ± 4.8^c^	0 ± 0^c^	0 ± 0^c^

A BC_4_ plant with the presence of *Rcr1* was self-pollinated to produce the Y549-(0)-2-1 breeding line homozygous at the *Rcr1* locus. DSI was determined through pathogenicity tests. Different letters indicate significant differences in DSI within each strain, as determined using ANOVA and post-hoc Tukey’s HSD test.

## Discussion

### *Rcr1* is an important clubroot-resistance gene

The recent publication of a reference *Brassica* A genome using one of the most valuable clubroot-resistance breeding resources, European fodder turnip ECD04 (*B. rapa* ssp. *rapa*, AA), has enabled us to obtain a broader view and deeper insights into the genetic and evolutionary basis of clubroot resistance in the A genome (Yang *et al.*, 2022). The study found that the 28 previously reported clubroot-resistance loci from the A genome could be physically mapped to 15 integrated loci in the ECD04 genome, allowing the previous clubroot-resistance loci groups of *CRa*/*Rcr1*/*Rcr2* and *CRb*/*Rcr4*/*Rcr5* to be integrated into new loci of *CRA3.7* (24.95–25.14 Mb on A03 of the genome) and *CRA3.4* (22.66–24.54 Mb on A03), respectively. In our current study, we have cloned and functionally verified the clubroot-resistance gene *Rcr1* (identified from pak choy cv. ‘Flower Nabana’, *B. rapa* ssp. *chinensis*), which is identical to *CRa* (identified from the Chinese cabbage line T136-8, *B. rapa* ssp. *pekinensis*) and *Rcr2* (identified from Chinese cabbage cv. ‘Jazz Napa Cabbage’) in the coding regions. This has confirmed that the identical clubroot-resistance gene *CRA3.7.1* (i.e. *BraA03g012133E* in ECD04) could be responsible for all of the clubroot-resistance loci (i.e. *Rcr1*, *Rcr2*, *CRa*, and *CRA3.7*) identified from this overlapped region. However, whether *BraA03g012133E* is the sole clubroot-resistance gene in this region is unclear because there are another four NBS-LRR (NLR)-type candidate disease-resistance genes present in this region of the ECD04 genome (Yang *et al.*, 2022), and the corresponding genomic regions in the other clubroot-resistant materials, such as pak choy cv. ‘Flower Nabana’ and the Chinese cabbage line T136-8, have not been fully sequenced or characterized. Although the high-quality genome of the European fodder turnip ECD04 has shown good prediction ability with the integrated clubroot-resistance locus of *CRA3.7*, there are still uncertainties as evidenced by the clubroot-resistance locus of *CRA3.4*. In this study, we have cloned the *Rcr1* gene from the clubroot-resistant turnip cv. T19 (harboring *Rcr4*) but not from the turnip cv. PTWG (harboring *Rcr5*). Besides, the clubroot-resistance gene *CRb-α* from the *CRb* locus has been found to be identical to *CRa* (Hatakeyama *et al.*, 2017). Therefore, the three clubroot-resistance loci (*CRb*/*Rcr4*/*Rcr5*) are not presumed to be integrated into the clubroot-resistance locus of *CRA3.4*. This uncertainty regarding the relationships among these three resistance loci is probably caused by the different clubroot-resistant materials studied and the uncharacterized pathotypes used for mapping (Hatakeyama *et al.*, 2017). Nevertheless, we have demonstrated that *Rcr1*, as a single dominant gene, is a potent clubroot-resistance gene showing a phenotype with a high level of resistance against strains of *P. brassicae* collected in western Canada (Table 5). Furthermore, its wide distribution in many important clubroot-resistant breeding materials might indicate an ancient role in the co-evolution of the *Brassica*–*P. brassicae* pathosystem (Table 1). Thus, *Rcr1* is confirmed to be a valuable genetic resource in the fight against clubroot disease.

### Intragenesis is advantageous for breeding for disease resistance

Transgenesis is cross-breeding executed at the molecular level, and the precise manipulation of a few genes using genetic engineering techniques has the potential to help obtain more specific breeding results at a much faster pace than those resulting from genome-wide recombination or random mutation. That is the basic logic for why transgenic crops have gained popularity so quickly worldwide following their first introduction. However, although no definite cases have been confirmed by scientists, the potential hazards such crops might present to humans and the environment have caused public concerns over safety, and hence strict regulations over transgenic crops have been imposed by governments worldwide. The constant and increasingly urgent need for crop improvement has therefore led to the search for alternative methods to address the key issues in the transgenic technique, namely the presence of an exogenous gene-of-interest and the unwanted residue sequences. Genome editing works on endogenous genes, and the unwanted residues can be removed through segregation. However, a prerequisite to developing genetically edited plants is the presence of endogenous genes or genetic elements in the same plant species coupled with a deep understanding of the functionality of the target genes, otherwise, the desired trait might have to be first introduced into the plant genome either using a transgenic approach or a site-specific insertion via genome-editing tools. Unfortunately, this prerequisite is not always available, especially for disease-resistance breeding for which adding exogenous genes rather than modifying endogenous ones is mostly the case. This reality warrants the niche of cis/intragenic plants in the field of crop improvement, particularly in disease-resistance breeding where introgressing resistance genes from allied species into an elite background is a common practice. Several surveys conducted in the European Union have shown that cisgenic plants are more acceptable to consumers than transgenic ones thanks to the native origin of the introduced gene-of-interest and the ‘clean’ genetic background (Gaskell *et al.*, 2010, 2011). In practice, many newly identified genes of interests are not yet fully characterized, including the functionality of the native regulatory elements, or their native regulatory elements are not desirable for specific breeding needs. In such cases, intragenesis is a better option compared with cisgenesis since using other well-studied promoters/terminators can accelerate the breeding process and, more importantly, help achieve a more predictable result. With regards to traditional introgression, although it has played and will continue to play an important role in disease-resistance breeding, it has struggled to keep up with the pace of fast-evolving pathogens such as the protist *P. brassicae* in clubroot (Yu *et al.*, 2017). As demonstrated in this current study, to obtain *Rcr1*-rendered clubroot resistance in canola-quality germplasms, traditional introgression breeding through interspecific crosses took at least 6 years compared to 2 years of breeding time using an intragenic method (Fig. 5B). This significantly increased breeding efficiency should help breeders to win the race against pathogens and to overcome other pressing challenges. In the past, the lack of cloned disease-resistance genes has limited the application of intragenic breeding; however, with the advent of the ‘omics era’, more and more resistance genes will be cloned and characterized, which should fuel the widespread adoption of this fast-breeding strategy. Moreover, if the desired trait/gene is derived from a relative plant species recalcitrant to cross-breeding, then introgression breeding is not even a viable option. Therefore, intragenic breeding can clearly be advantageous over other options for disease-resistance breeding.

### The modified intragenic vectors and workflow should work well for future intragenic plant breeding

*Rcr1* is a single dominant clubroot-resistance gene encoding a TNL type of protein, but its full functionality including regulatory features of the original promoter/terminator is not yet fully characterized. To develop canola germplasms carrying this newly identified clubroot-resistance gene, we have modified the pHHCGS vector system (Hu and Yu, 2022) into pHHIGS vectors for intragenic breeding, since genome editing and cisgenesis are not suitable for this specific need. In the pHHCGS vectors, the key feature of auto-excision of the unwanted residue sequences is realized by the embedded multi-clonal sequence (Supplementary Fig. S3), which comprises three copies of targeted sequences for CRISPR/Cas9 recognition and editing, and this feature was fully retained in the pHHIGS vectors (Fig. 1A). As reviewed by Schmidt *et al.* (2019), the three incidents of CRISPR/Cas9-induced double-strand breaks on the same chromosome successfully induced heritable deletions of the flanked region in canola. The targeted sequence in the embedded multi-clonal sequence was carefully designed so that it has the lowest homology hit with major crops (such as *Brassica* crops) via NCBI blasting (https://blast.ncbi.nlm.nih.gov/Blast.cgi), as well as the lowest off-target score in the *B. napus* background (Genoscope, V5) via CRISPR-GE prediction (http://skl.scau.edu.cn/) (Xie *et al.*, 2017). The off-target mutation is critical in evaluating CRISPR/Cas-based applications (Chen *et al.*, 2019), and the fact that no off-target mutations could be detected in our study using an extended list of predicted off-target sites demonstrated the outstanding performance of the pHHIGS vectors in the *B. napus* background (Table 4). Nevertheless, when applied in plants outside the *Brassica* genus, it is strongly advised to check the specificity of the targeted sequence (Hu and Yu, 2022) against the intended genomic background. We employed different promoters for *Cas9p* (*AP1*, *CLV3*, and *Hsp18.2*) to provide more control over the editing activity (Hu and Yu, 2022). Since heat treatment has been reported to affect CRISPR/Cas9-mediated gene editing activities (LeBlanc *et al.*, 2018; Hu and Yu, 2022), we characterized the synergetic effects of a heat treatment and the different promoters on the editing activities of the modified intragenic vectors in the canola background. As shown in Fig. 4B, the editing efficiency was generally acceptable (~20%) when the respective stimulus (i.e. developmental cues or heat treatment) was applied, and 100% efficiency was achieved when using pHHIGS-Hsp18.2 when two heat episodes were applied (‘HT1 + 2’). The moderate editing efficiencies observed for certain combinations of vector and treatment should not be a concern since screening is part of the breeding process, and even 20% of edited plants with single-copy inserts and heritable deletions could produce enough desirable progenies in the next generation. The varying editing efficiencies observed in this study can most likely be attributed to co-ordination between the heat treatment and the sgRNA/Cas9p complex. The expression level of sgRNA was almost constant (~1.5-fold that of *BnACTIN2*) throughout the monitored period across the different treatments (Fig. 4C), suggesting that the abundant sgRNA driven by the *AtU6-26* promoter was not a bottleneck for editing activities in all the cases studied. In contrast, the varying expression of *Cas9p* would be partially responsible for the significantly different editing efficiencies observed (Fig. 4B, C). Further, heat treatment at 37°C has been found to increase the activity of Cas9 nuclease (LeBlanc *et al.*, 2018), which would explain the significant increase in the editing efficiencies between ‘HT1 + 2’ and ‘No HT’ treatments for both the vectors pHHIGS-AP1 and pHHIGS-CLV3 (Fig. 4B) where no significant changes in *Cas9p* expression were observed (Fig. 4C). Although this current vector system used the minimum design for the core sequence of the embedded multi-clonal sequence, there will be some residue sequences left even after successful and complete editing/cleaning, which will include T-DNA borders, the unused restriction sites, and the remnants of the cutting sites (Fig. 1A). These residues are by nature non-coding, and thus are unlikely to cause phenotypic effects in the final product (Schouten *et al.*, 2006b). If bacterial T-DNA borders become a major concern, the T-DNA in our vector system could be replaced by P-DNA, which are DNA sequences naturally present in plants that are essentially identical to T-DNA and can be used to functionally mimic T-DNA borders (Rommens, 2004).

With the first CRISPR/Cas9-based vector system for intragenic breeding, we have designed a streamlined workflow that is fast and easy to use, and it should be capable of being seamlessly applied to other crops that can be transformed via *Agrobacterium* (Fig. 1B). The workflow has four steps, as follows. Step 1: construct the custom pHHIGS vector via restriction cloning using the promoter/terminator derived from the same or sexually compatible species of the recipient plant and directly insert the coding sequence of the gene-of-interest into the vector. Step 2: after *Agrobacterium*-mediated transformation, use ddPCR to screen for T_0_ transformants with a single-copy insert of T-DNA. Step 3: after induction of genome editing, use PCR to select edited T_0_ plants and further confirm successful deletion using Sanger sequencing. Step 4: conduct further selection on the T_1_ generation for progenies that are homozygous for the gene-of-interest and have no abnormal phenotypes. As demonstrated in our study, following this workflow can lead to the development of intragenic plant germplasms with a homozygous gene-of-interest within two generations for a sexually propagated plant, such as canola. For asexually propagated plants, further selection at Step 4 should be conducted on the vegetatively propagated materials, and thus intragenic germplasm could be generated within the T_0_ generation.

### Conclusions

In this study, *Rcr1* was first cloned and verified for its clubroot-resistance functionality, and it was then incorporated into an elite canola line using a modified CRISPR/Cas9-based intragenic vector system, which resulted in a fast breeding strategy. Following a streamlined workflow, selection-marker-free canola germplasms with potent *Rcr1*-rendered clubroot resistance against multiple representative Canadian strains of *P. brassicae* were successfully developed. The whole breeding process only took ~2 years, which was much shorter compared to the 6 years needed using traditional introgression. The design of the intragenic vector system and workflow can easily be applied to other crops that can be transformed via *Agrobacterium*, and this technique should be useful for other similar applications in molecular plant breeding in the future.

## Supplementary data

The following supplementary data are available at *JXB* online.

Table S1: Primer and probes used in this study.

Fig. S1. Sequences of the three different promoters.

Fig. S2. Sequence alignment of *CRa* from Chinese cabbage versus *Rcr1* from pak choy.

Fig. S3. The embedded multi-clonal sequence in the pHHCGS vectors.

erad471_suppl_Supplementary_Tables_S1_Figures_S1-S3

## Data Availability

All data supporting the findings of this study are available within the paper and within its supplementary materials published online. The vectors are available from the corresponding author, Fengqun Yu, on a reasonable request.
