# Supplementary material for: A CRISPR/Cas9-based vector system enables the fast breeding of selection-marker-free canola with Rcr1-rendered clubroot resistance
Source: J Exp Bot. 2023 Nov 22;75(5):1347–63. doi: 10.1093/jxb/erad471 (PMC10901203; doi:10.1093/jxb/erad471)
Supplement: erad471_suppl_Supplementary_Tables_S1_Figures_S1-S3 [file erad471_suppl_supplementary_tables_s1_figures_s1-s3.pdf]

Supplementary Table S1. Primers and probes used in this study.

Primer name	Sequence (5'-3')	Note
**caccRcr1F**	CACCATGGATTTCTCTCTTTTCCTTACCATCG	Gateway cloning primers for *Rcr1* to work with pENTR-D-TOPO vector
**Rcr1R**	TTAACATGAGGGAGTTTCCAGAGGATG	
**SbfI-Rcr1F**	AGTCCCTGCAGGATGGATTTCTCTCTTTTCCTTACCATCG	Cloning primers for *Rcr1* to work with pHHIGS vectors
**MluI-Rcr1R**	AGTCACGCGTTTAACATGAGGGAGTTTCCAGAGGATG	
**F**	GCAAGTTAAAATAAGGCTAGTCCG	PCR confirmation on the entire flanked region; 189 bp of amplicon after editing
**R**	GGATGTGCTGCAAGGCGATTAAG	
**BnActin2-P**	HEX-TGCTGGATTCTGGTGATGGTGTGT-BHQ1	Insert copy number screening by ddPCR, or for the relative expression level of *Cas9p*/sgRNA by RT-qPCR; *BnActin 2* as reference gene
**BnActin2-F**	CAGTGGTCGTACTACTGGTATTG	
**BnActin2-R**	GATGGCGTGTGAAAGAGAGA	
**Bar-P**	6-FAM-TCATGCCAGTTCCCGTGCTTGAA-BHQ1	Insert copy number screening by ddPCR; *Bar* as the target gene
**Bar-F**	AAGTCCAGCTGCCAGAAA	
**Bar-R**	CGAGGCGCTCGGATATG	
**Rcr1-P**	6-FAM-GCTGGTTAATTCTCCACAACCGAGTC-BHQ1	Insert copy number screening by ddPCR; *Rcr1* as the target gene
**Rcr1-F**	CTAGGGATGATGAAGCTACCGTG	
**Rcr1-R**	CATCCTCACTTCCTTGGAGCC	
**BarPTC-F**	CACCATCGTCAACCACTACATCG	Post-transformation confirmation for *Bar* gene by PCR; 422 bp of amplicon
**BarPTC-R**	AACCCACGTCATGCCAGTTC	
**Cas9pPTC-F**	GGTGCCTAATGAGTGAGCTAACTCAC	Post-transformation confirmation for *Cas9p* gene by PCR; 1082 bp of amplicon
**Cas9pPTC-R**	CGTCCACTCCTGCGGTTC	
**Rcr1PTC-F**	GGATGATGGCATATGCAGCAGC	Post-transformation confirmation for *Rcr1* gene by PCR; 789 bp of amplicon
**Rcr1PTC-R**	GCGGACAAACCAGTTGGATAAATGG	
**Cas9RT-F**	CGCTCAGATTGGAGATCAGT	Quantification of *Cas9p* gene expression by RT-qPCR; from Ma et al., 2015
**Cas9RT-R**	CCTGGTGGTGCTCGTCGTAG	
**sgRNART-F**	GACCATCGACACCTAGTGACG	Quantification of sgRNA expression by RT-qPCR; from Ma et al., 2015
**sgRNART-R**	CGACTCGGTGCCACTTTTTCAAGTTG	

Supplementary Fig. S1. Different promoters

Hsp18.2 720bp

ATGGTCATTTCTTCTGGTTCAAGCATGACATGAACAGGCAATAAATAAGTTGAGATTTTGAT
CACAGTAACTGATACTTGAATCGAATCATTTAGATTTTTTTTTTTTTTAGTTTACTTGTTTAGT
AAATATGTTGTCTATGTTTGTCACAAAAACGTGGCTCAGTTCTTGTATATATGGAGACAAAA
AAATCCATTAAAAGATTGTTGACATTCTCGGAAATTTAGTGCCAACTGTTATTGCGAGAACT
TACTATAGTTTTCCTTTGGCGAAAAGCTAATAATCTTAAATCTTGATTTTGTCCTCTTTTCTCT
GAGTTAGATTTTCTTAAATTCCACTTCCGACCTATTAAGAAATGGGCTTTTGCAAAGAAGAT
CCGCTTCACTGAGCCCGTATCTCGAAGAGGATAATACAACAACAAAGCAAAACGGCACGTA
GTTTTAATTGTAACCAAGGATTGCATTTCGGTCTTGTTTCAACAAACGAAACTTCCTGAAATG
CCAAGAAAAATCTGGTCATTTCACCACAGTGATCATTGTGTATGTGTTCTAAAGACTCCAAG
CGAAGGTTTTAGAAAAAGGAGCATTTTCTATTCTATTCAAGAAACTCGAAGAACATTCTCTC
TTCATCCTCTAACTTCCCTATAAATATGTCCTTTGCTAATCAGATCAAATCAGCAGGAAAATC
AAGAACCAAAAGTCTCCCGAAAAGCAACGAACA

CLV3 1444 bp

CGGATTATCCATAATAAAAACAAAACTAGATACATTTTCTAAGTACATTAACACATAAGAAT
ATCATTTTTGTACTACCAAAAAAAAAGTAAAAACTTATGTCCATGGTATTATTATAATTGGT
GGTAAATCAGTAAATTAGTTGTTAGCAAAAAAATAAAATTTAGTTAAAAAGTAGTGGCACCT
TATTGGCCCAAAAGAGTAACGAAGCAAAACGGAATCTGAAAACTGTTAAAACTTAAAAGAA
TAGATAATTGAGATATAATATCGTATGATCGGACGGCTGTGATCTGATGCCATCGGATGGGC
ACGTGTCAGAAGTGTACTCCAGGTATCATTCTCTCTGCCTCATGACATCAGCCGATGGTACC
CTCGATACGGCGCCGGCAAGGCTCATATAATCCATTCAATTTATGTTTTTTCTGCTAGCAAAT
AGATTTGCCATTGATTTGATACTCTCAATAATATTATCCGATTAAGGTATCATCCATATTGTT
CAATATAATTTAAGCATATAACTGTTTCCAGATTAAACAATATAATTTATAAGAGCAACTGT
AATACTTTACTTTAAAAGTTTTCAAATCAGAATCTCTTTTCTTTTTCTACAAATCTGGAAACA
ATTTAATCCATATTGTTTTTGTTTTTTTTACCTTCTCATATTTAGATGCTATTTATTATTGTGAC
ACAAGCTTAGTTTAATTTCTTGTTGGTTACATTTTTTTTTCTAAATACACAAATATTATATGTT
TAATATTATTTCACACATTTCTTAGAAGAAATCTAAATACATGAAATTATATTTATTAAAAAG
AAGTCTAAACTATATATGTATTTAAATGGAATTTTAAATATGGGTAGTAATATAGAAACACC
ATTGATATATTAGAGTATGTGCCGGTGCCGTATACATACTGTTGTATTATAGTAACCTATATA
TGGTATACCTAGTCAATGTGTACGGTATTTTTCATATGTATTAGTTGTGAACCTCCACAGCAT
GTTAGACTTAGGAATTAATTATAACTGAACCAGAACTTTGAGTCTAATTATACCCCTGCCGTT
TTATACAACTTTGAATATTGATTCCAGTGGCTAATTTAAATAAGTTAAAGGAAACAAAAACT
GCAAGTAAGTGAAAAATACACAATTGTAAGTAAATGTTAGATATTTAATTTATTTATAGCTA
AATCATGAACAAGTTCGTATAAGATCTAGATATATGTACCATATACCTTTTCTATGCCCACTA
TATATACTTACTACACATAATATATAAACCAACCAAAATATTTGAATAGTTAATAACTATGA
TACACGTTTAGGACAAATAAAATTAAAAAATAGGAGATCCCATTTCTCGCCCTTGTAGGCTT
ACGCTATAAATTGGACTGTCCCCTTCTCATTTCATTACCAAAGTAAAGAACAGTTTCTATATT
TCTCT

AP1 1895 bp

CTTGGGATGTTGTCTTCAAGGCCACGAGCTTAGATTCTTTTAGTTTTGCTCAATTTGTTAAGT
TTCTACTTTTCCTTTTGTTGCTTACTACTTTTGCTCATGATCTCCATATACATATCATACATAT
ATATAGTATACTATCTTTAGACTGATTTCTCTATACACTATCTTTTAACTTATGTATCGTTTCA
AAACTCAGGACGTACATGTTTAAATTTGGTTATATAACCACGACCATTTCAAGTATATATGT
CATACCATACCAGATTTAATATAACTTCTATGAAGAAAATACATAAAGTTGGATTAAAATGC

AAGTGACATCTTTTTAGCATAGGTTCATTTGGCATAGAAGAAATATATAACTAAAAATGAAC
TTTAACTTAAATAGATTTTACTATATTACAATTTTTTCTTTTTACATGGTCTAATTTATTTTTCT
AAAATTAGTATAATTGTTGTTTTGATGAAACAATAATACCGTAAGCAATAGTTGCTAAAAGA
TGTCCAAATATTTATAAATTACAAAGTAAATCAAATAAGGAAGAAGACACGTGGAAAACAC
CAAATAAGAGAAGAAATGGAAAAAACAGAAAGAAATTTTTTAACAAGAAAAATCAATTAGT
CCTCAAACCTGAGATATTTAAAGTAATCAACTAAAACAGGAACACTTGACTAACAAAGAAA
TTTGAAACGTGGTCCAACTTTCACTTAATTATATTGTTTTCTCTAAGGCTTATGCAATATATG
CCTTAAGCAAATGCCGAATCTGTTTTTTTTTTTTTTGTTATTGGATATTGACTGAAAATAAGG
GGTTTTTTCACACTTGAAGATCTCAAAAGAGAAAACTATTACAACGGAAATTCATTGTAAAA
GAAGTGATTAAGCAAATTGAGCAAAGGTTTTTATGTGGTTTATTTCATTATATGATTGACATC
AAATTGTATATATATGGTTGTTTTATTTAACAATATATATGGATATAACGTACAAACTAAATA
TGTTTGATTGACGAAAAAAAATATATGTATGTTTGATTAACAACATAGCACATATTCAACTG
ATTTTTGTCCTGATCATCTACAACTTAATAAGAACACACAACATTGAACAAATCTTTGACAA
AATACTATTTTTGGGTTTGAAATTTTGAATACTTACAATTATTCTTCTCGATCTTCCTCTCTTT
CCTTAAATCCTGCGTACAAATCCGTCGACGCAATACATTACACAGTTGTCAATTGGTTCTCA
GCTCTACCAAAAACATCTATTGCCAAAAGAAAGGTCTATTTGTACTTCACTGTTACAGCTGA
GAACATTAAATATAATAAGCAAATTTGATAAAACAAAGGGTTCTCACCTTATTCCAAAAGAA
TAGTGTAAAATAGGGTAATAGAGAAATGTTAATAAAAGGAAATTAAAAATAGATATTTTGG
TTGGTTCAGATTTTGTTTCGTAGATCTACAGGGAAATCTCCGCCGTCAATGCAAAGCGAAGG
TGACACTTGGGGAAGGACCAGTGGTCCGTACAATGTTACTTACCCATTTCTCTTCACGAGAC
GTCGATAATCAAATTGTTTATTTTCATATTTTTAAGTCCGCAGTTTTATTAAAAAATCATGGA
CCCGACATTAGTACGAGATATACCAATGAGAAGTCGACACGCAAATCCTAAAGAAACCACT
GTGGTTTTTGCAAACAAGAGAAACCAGCTTTAGCTTTTCCCTAAAACCACTCTTACCCAAAT
CTCTCCATAAATAAAGATCCCGAGACTCAAACACAAGTCTTTTTATAAAGGAAAGAAAGAA
AAACTTTCCTAATTGGTTCATACCAAAGTCTGAGCTCTTCTTTATATCTCTCTTGTAGTTTCTT
ATTGGGGGTCTTTGTTTTGTTTGG

Supplementary Fig. S2. Sequence Alignment *CRa* (T136-8) vs. *Rcr1* (FN, initial amplification)

```
Query=CRa(T136-8) Length: 4609
Sujct=Rcr1(FN, initial amplification) Length: 4619
Score:8282 bits(9184), Expect:0.0,
Identities:4601/4609(99%),  Gaps:0/4609(0%), Strand: Plus/Plus
```

(Note: scores cited from NCBI website; different sequences are noted with red fonts; framed sequences were PCR primers used to amplify Rcr1/CRa)

Rcr1F

```
Query  1     ATGGATTTCTCTCTTTTCCTTACCATCGTTGCTGCTGCAATAGGTTTCTTCGTGATTCGA  60
Sbjct  1     ........A...A.........A.....................................  60
Query  61    AGATTCAGATTCAATCCAGAAAACAATGAAATCGATACTTCGTCTTTGTCTCCATCATCA  120
Sbjct  61    ............................................................  120
Query  121   CCACCATCTTCTTTATCTTCTAGGTCTTTGTCTACATCATCAACTCCATCTGCTTTGTCT  180
Sbjct  121   ............................................................  180
Query  181   CCTTCATCGGCTTCTCCATCTTCTTCCTCTCATGTCTGGATGTACGATGTCTTTCCGAGC  240
Sbjct  181   ............................................................  240
Query  241   TTCCGTGGGGAAGATGTCCGCTACAACTTTCTCAGTCACATTAAAAAGGAGTTTAAAAGG  300
```

```
Sbjct  241   ............................................................  300
Query  301   AAGACAATCACATTTTTCAACGATAATGGGATCGAGAGAGGAGAATCCATCGCTCCTGAA  360
Sbjct  301   ............................................................  360
Query  361   CTCATACAGGGGATTAGAGGATCTAAGATTGCGATCGTCTTGCTCTCTACGAACTACGCT  420
Sbjct  361   ............................................................  420
Query  421   TCTTCAAAGTGGTGTCTTGAGGAGTTGGTGGAGATTATGAAGTGCAGGGAGGAGCTTGGT  480
Sbjct  421   ............................................................  480
Query  481   CAAACTGTGATTGCTATTTTCTACAAAGTAGATCCATCTGACGTAAAGAAGCTGACCGGA  540
Sbjct  481   ............................................................  540
Query  541   GACTTTGGGGAGGTTTTCAGAAAGACTTGTAAGGGTAAAGCTAAGGAGGAGATTAGGAGG  600
Sbjct  541   ............................................................  600
Query  601   TGGGAACAAGCTTTGGAGAAGGTGGCCGTAATAGCTGGTTACCATTTATCCAACTGGTTT  660
Sbjct  601   ............................................................  660
Query  661   GTCCGCTTAACTTTTACTTGCTATCTTTCAAACACATCCACAACATATGATATGTATAGA  720
Sbjct  661   ............................................................  720
Query  721   AAAAATACTCAAAATATGAAACAAAAATAAACAAAATTTTCAAAAATATGACAAGAGTTT  780
Sbjct  721   ............................................................  780
Query  781   AGGTTTACGAAAGAGTCCATAAAAGTTTACTACGGTCTCCTAAAATTTTAGAAACGTAAT  840
Sbjct  781   ............................................................  840
Query  841   AAAGTTGTGGCGTTTTCAAATTTTTATGATTTCGGTTATAATTTTGGAACAGAAAAAATG  900
Sbjct  841   ............................................................  900
Query  901   TAAGTTCTTCCATTTTTCTAGTCACGTAATGTGAAAGTTATACATGCTTAATTTTTATAC  960
Sbjct  901   ............................................................  960
Query  961   CAAAATTAAAAATAGTAAACTTTTATCATCTCTGTATCAATTTTGTCTCTGATTGTGTGT  1020
Sbjct  961   ............................................................  1020
Query  1021  AGTAAATTTAATGACTATATAGTTGATGTTTTCTTCTCTCCTCTTTCTAGGGATGATGAA  1080
Sbjct  1021  ............................................................  1080
Query  1081  GCTACCGTGATCGAGAATATATCAACATGTGTTTTAAACAAGCTGGTTAATTCTCCACAA  1140
Sbjct  1081  ............................................................  1140
Query  1141  CCGAGTCATTTCGACAATCTAGTTGGGATGAGTACTCATATGGAAAATCTGGAACTGTTG  1200
Sbjct  1141  ............................................................  1200
Query  1201  TTAAGCCTGGGCTCCAAGGAAGTGAGGATGGTAGGGATTTGGGGTCCTTCTGGAATTGGT  1260
Sbjct  1201  ............................................................  1260
Query  1261  AAGAGCACCATCGCCAGAGTTCTATTCAACCAACACTCTCATCAGTTCCAATTTAGTGTC  1320
Sbjct  1261  ............................................................  1320
Query  1321  TTTATGGAGAACATTAAAAGACTTTGGCCCAGACCTTATTACGATGAGTACAGTGTGAAA  1380
Sbjct  1321  ............................................................  1380
Query  1381  CTGCAACTACAAGAAGAGTTCCTGTCCCGGGTAATCAACCAAAAAGATATCAAGATTCAG  1440
Sbjct  1381  ............................................................  1440
Query  1441  CAATTAGGAGTTGTGGAAGACAGGTTAAAGGACAAGAGAGTGCTTGCCATTCTTGATGAC  1500
Sbjct  1441  ............................................................  1500
Query  1501  GTGGATCATTTGTTGCAAATAGAAGCCATAGCTAAAGAAGCTCGGTGGTTTGGTCCTGGA  1560
Sbjct  1501  ............................................................  1560
Query  1561  AGTTGGATAATCATCACAACACAAGATAAAAGGCTTTTATATGCACATGGAATTAACCAG  1620
Sbjct  1561  ............................................................  1620
Query  1621  ATTTACGAGGTGGAGCTTCCACCTGACGAAGAGGCTCTTGAAATTTTCTGCATGAATGCT  1680
Sbjct  1621  ............................................................  1680
Query  1681  TTTTGTCAAAAATCTCCACCTGATGGCTTCAAGGAACTTGCCTGGGAAGTTACAAGACTT  1740
Sbjct  1681  ............................................................  1740
Query  1741  GCAGGTAAGCTCCCTTTGGGACTGAGGGTTATGGGATCTCATTTCAAAGGAAGGCCCAAG  1800
Sbjct  1741  ............................................................  1800
Query  1801  CATGAATGGGAAGAGGGACTACCAAGGTTAAGAACTAGACTTAATGGAGAAATTGAAAAT  1860
Sbjct  1801  ............................................................  1860
Query  1861  ACTTTAAAGTTCAGTTATGATGCCTTATGCGATGACAATCAAGCTATATTTCTTCACTTA  1920
```

```
Sbjct  1861  ............................................................  1920
Query  1921  GCATGCTTTTTCATCAATGAGCCGATTGAAAACGTGGAACGGTGTCTTGAAAAAAAAATT  1980
Sbjct  1921  ............................................................  1980
Query  1981  GTTGGTGTGAAAGGTTGTCTTCGTGTTTTAGCTGAGAAATCTTTCATATCCTTTGAGTGG  2040
Sbjct  1981  ............................................................  2040
Query  2041  GGACGTATAAAGATGCATGATTTGCTAGCACTTTTGGGTAGAGAAATTGTTCGTAAACAA  2100
Sbjct  2041  ............................................................  2100
Query  2101  TCCATTCATGAACCTGGGCAGCGTCAGTTTTTGGTTGATGCTGGAGATATATGCCAAGTA  2160
Sbjct  2101  ............................................................  2160
Query  2161  CTACGAAACGATACACTAGTAAGTTTTCACATTAGTCATTTAATGCATTTCTCCCTATAA  2220
Sbjct  2161  ............................................................  2220
Query  2221  AAATCATATTTGTTTGTGTATCGTTGGGTTCTTTTGCAGGGTAGTCGAAATGTTATAGGC  2280
Sbjct  2221  ............................................................  2280
Query  2281  ATAGATTTGGACCTTACAAAGTTGGAGACGGAGGTGAAGATAAGTGACAGAGTTTTTGAA  2340
Sbjct  2281  ............................................................  2340
Query  2341  AGAATGCCCAATGTCCAATTCTTAAGAGTCAAATATAGAAGTATTCAGAGAAAGCCATAT  2400
Sbjct  2341  ............................................................  2400
Query  2401  CCTCACAGCATAGATCCCGTGACATGTCTGCCCCCAAATCTAATTATCCTGCATTGGGAT  2460
Sbjct  2401  ............................................................  2460
Query  2461  TATTTTCCGATGACATGTCTCCCTTCTAATTTTAATCCGGAGTTCCTGACGAGAATAATC  2520
Sbjct  2461  ..............T.............................................  2520
Query  2521  TTGACTGAAAACAACTACCTTGAGAAATTGTGGGAAGGAAATAAAGTAAGTAAAATTTGT  2580
Sbjct  2521  ............................................................  2580
Query  2581  ACTTTTTATTCGAAATTATAATTTGTCTTTCTGTGCATATACGTTAGCTGAAAAAGGTTA  2640
Sbjct  2581  ............................................................  2640
Query  2641  TCTTGTCTTAACGGACCGTCTTCTTTTATTGATATTTTTCCCCCCtttttttCCTGTACA  2700
Sbjct  2641  ............................................................  2700
Query  2701  GACGATTAGAAATCTGAAATTGATGAATTTGTCGAACTCCAAAAATCTAAAGGAGCTTCC  2760
Sbjct  2701  ............................................................  2760
Query  2761  CGATCTCTCAACTGCCACTAATCTGCAAACATTGGAGCTTTCTGGTTGCTCAAGTCTTAC  2820
Sbjct  2761  ............................................................  2820
Query  2821  GGAACTCCCCTTTTCTATTGGAAATGCTATCAATCTCCGGCGTTTGAATCTTAGTCATTG  2880
Sbjct  2821  ............................................................  2880
Query  2881  CTCAAGTCTGATGGAACTCCCCTCTTCTATGGAAAATGCCACTGATCTCGAGGAACTGAA  2940
Sbjct  2881  ............................................................  2940
Query  2941  TCTCACGGGATGCTTACATCTAGCTAAGCTCCCATCCTCTATTGGTAATCTCAAGAAATT  3000
Sbjct  2941  ............................................................  3000
Query  3001  GTATCTCAAAGATTGCTCAAGTTTGGTGGAGTTCCCCTCTTCTATGGAAAATGTCACTAC  3060
Sbjct  3001  ............................................................  3060
Query  3061  TCTCGAGGAATTGCTTCTCACTGGATGCTCACATCTAGCTAATCTCCCACCTTCTATTGG  3120
Sbjct  3061  ............................................................  3120
Query  3121  TAATCTCAAGACGTTGTATCTCGAGAATTGCTCAAGTTTGGTGGAGCTTCCATCTTCTGT  3180
Sbjct  3121  ............................................................  3180
Query  3181  TAGAAATTCCATTAATCTCAAGAATTTTTCTTTTAATGGTTGCTCAAATCTTGTGGAGCT  3240
Sbjct  3181  ............................................................  3240
Query  3241  CCCTTTCTATCTTGGTAATGCCACTGATCTCCAGAGATTGTATTTGAGAGGATGTTCAAG  3300
Sbjct  3241  ............................................................  3300
Query  3301  TCTACAGGAGCTTCCTTCTTCTATTGGGAATATAACTCGTCTTGAGGAGTTGATTCTCGA  3360
Sbjct  3301  ............................................................  3360
Query  3361  AGAATGCTCAAGTCTTGTTGAGCTCCCTTCTTCTATCGGAAATATAACTAGTCTCGAGTA  3420
Sbjct  3361  ............................................................  3420
Query  3421  CTTGAATCTTGATGCGTGTTCAAGTCTTGTGAAGCTCCCCTCTTCTATTGGAGATATAAT  3480
Sbjct  3421  ............................................................  3480
Query  3481  CAATCTGAAGAACTTGTATCTTAATGGATGCTCAAGTCTTGTGGAACTCCCATCTTCTAT  3540
```

```
Sbjct  3481  ............................................................  3540
Query  3541  TGGAAATATAAATTATCTCAAGAAGTTGTCTCTTAATGGATGCTCAAGTCTGGTAGAGCT  3600
Sbjct  3541  ............................................................  3600
Query  3601  CCCCTCTTCCATTGGGAATATGACTAGTCTTGAAGAGTTGAATCTTAATGGATGCTCAAG  3660
Sbjct  3601  ............................................................  3660
Query  3661  TCTTGTGGAACTTCCATCTTCTATTGGAAATATGAATAATCTCTGGATGTTGTATCTCGA  3720
Sbjct  3661  ............................................................  3720
Query  3721  AAGATGCTCCAACCTAACGGCCCTTCCGATCAATATCAACATGAAATCTCTTCGTGTACT  3780
Sbjct  3721  ............................................................  3780
Query  3781  TGCTCTCACAGACTGCTCGTCATTGAAAAGCTTTCCGGAGATTTCCACAAACATCAGAGT  3840
Sbjct  3781  ............................................................  3840
Query  3841  TCTAAAGCTCACTGGAACTGCAATAGAAGAAATCCCTCCATCAATCATGTCATGGCCATG  3900
Sbjct  3841  ............................................................  3900
Query  3901  GCTTTCTGAGTTAAATATGTCATACTTGGAGAACCTCAAGAAATCTCAGCATGCTTTATA  3960
Sbjct  3901  ........G...................................................  3960
Query  3961  CCGCATCACGGACCTGCTTTTGAGTGATACAGGAATACAAGAAACTGCTCCATGGGTCAA  4020
Sbjct  3961  ............................................................  4020
Query  4021  GAAGGAAAGGTCTCGTATACGTGAACTTGTAATCAAGCGATGCACTGAGCAAGTTTCTTT  4080
Sbjct  4021  ............................................................  4080
Query  4081  TCCACAGGATTGTAAGTTCTGCAAGAGACATCATTTCTCTCTTTACAAGCACAAGTCTAA  4140
Sbjct  4081  ............................................................  4140
Query  4141  TGTGTTCATCGTTATCAACTGCCTTAAACTGAATCAAGAAACAAGAGACCTTATCATCAA  4200
Sbjct  4141  ............................................................  4200
Query  4201  GACATCGACAAGAGACTTTACAATTCTGCCCGGAGAAACAGTGCCCACATATTTCAGTTA  4260
Sbjct  4201  ............................................................  4260
Query  4261  CCGAGCCGCCGGGAGTTCCCTGTCAATGACGTGGAATGGATTAGATACAGAGTACTTTCC  4320
Sbjct  4261  ............................................................  4320
Query  4321  TACATCCTTGAGATTTAGAGCTTGCCTCTTGCTGGTTTATAAGGGTCACGTTGGTGGTCA  4380
Sbjct  4321  ............................................................  4380
Query  4381  TCGGCAGTGGTCTGAAATAACTTATTGCATCAAGGACAAGCTCACTGGTGTTGAACGTTA  4440
Sbjct  4381  ............................................................  4440
Query  4441  TTCTCATAGATTTGTCAACTTGCCTCCAACTCCAGACGACCATCTGTTCGTATTCGAAAT  4500
Sbjct  4441  ............................................................  4500
Query  4501  TGAAGAAACTGTGAGTGCTCCTGAGTTAGTCTTTGAGTTTGGATTCACCAACAAAAATTG  4560
Sbjct  4501  ............................................................  4560
Query  4561  GGAGATTAAAAAATGCGGGCTACATCCTCTGGAAACTCCCTCATGTTAA 4609
Sbjct  4561  ...........................................C...CATGGAAGCTGA 4619
```

Rcr1R

Supplementary Fig. S3. Embedded multi-clonal sequence (EMCS)

agtcACTAGTGACCATCGACACCTAGTGACAGGGGCGCGCCGACCATCGACACCTAGT
GACAGGCCTAGGCCTGTCACTAGGTGTCGATGGTCTTAATTAAgtac

Note: 20 bp of the target sequence in yellow highlight, 3 bp of PAM sequence in green font.
